# Context-Aided Sensor Fusion for Enhanced Urban Navigation

**DOI:** 10.3390/s121216802

**Published:** 2012-12-06

**Authors:** Enrique David Martí, David Martín, Jesús García, Arturo de la Escalera, José Manuel Molina, José María Armingol

**Affiliations:** 1 Applied Artificial Intelligence Group, Universidad Carlos III de Madrid, Avda de la Universidad Carlos III 22, 28270 Colmenarejo, Spain; E-Mails: jgherrer@inf.uc3m.es (J.G.); molina@ia.uc3m.es (J.M.M.); 2 Intelligent Systems Lab, Universidad Carlos III de Madrid, Avda de la Universidad 30, 28911 Leganes, Spain; E-Mails: dmgomez@ing.uc3m.es (D.M.); escalera@ing.uc3m.es (A.E.); armingol@ing.uc3m.es (J.M.A.)

**Keywords:** autonomous navigation, multi-sensor fusion, intelligent systems, context exploitation, urban navigation

## Abstract

The deployment of Intelligent Vehicles in urban environments requires reliable estimation of positioning for urban navigation. The inherent complexity of this kind of environments fosters the development of novel systems which should provide reliable and precise solutions to the vehicle. This article details an advanced GNSS/IMU fusion system based on a context-aided Unscented Kalman filter for navigation in urban conditions. The constrained non-linear filter is here conditioned by a contextual knowledge module which reasons about sensor quality and driving context in order to adapt it to the situation, while at the same time it carries out a continuous estimation and correction of INS drift errors. An exhaustive analysis has been carried out with available data in order to characterize the behavior of available sensors and take it into account in the developed solution. The performance is then analyzed with an extensive dataset containing representative situations. The proposed solution suits the use of fusion algorithms for deploying Intelligent Transport Systems in urban environments.

## 1. Introduction

The evolution towards advanced Intelligent Transportation Systems (ITS) is usually based on the appearance of new operational concepts, supported by the state of the technology and their capability to implement critical functions with certain required performance. For instance, in the case of air traffic management, the enabling technologies to increase safety and efficiency of airspace have been surveillance systems (providing traffic situation to control centers) and navigation (letting the aircraft follow the desired route within maximum error bounds) [1]. Navigation technologies make vehicles capable of determining their position and velocity with respect to a certain reference, an essential aid to let them follow the desired routes with appropriate control techniques to enable autonomy. The information about position, kinematics and heading is required to be very precise and reliable in the new Intelligent Transportation Systems [2].

The development of automation for Intelligent Vehicles has received a noticeable attention of researchers in the areas of improved navigation systems, perception and autonomy. The DARPA initiative [3] pushed researchers to develop advanced and robust solutions in the way towards driverless cars. The first challenge in 2005 called for multi-sensor integration to allow navigation and terrain mapping to adapt driving to the conditions. Later, the urban challenge in 2007 [4,5] required more intelligent skills like following traffic rules or coordinated maneuvers with other actors in urban environments. The motivation has been pulling diverse sensing and information processing technologies to solve complex situations and promoting the achievement of common social benefits such as reduction of accident rates, sustainability by reduction of fuel consumption and traffic jams or increase comfort in driving.

Some of the main areas where new technological tools are expected are traveler information systems, incident prevention, driver assistance [6] and cooperative driving [7] including cooperative collision warning systems (CCW) [8]. In all of these new applications, a key issue will be to provide increased performance of navigation systems, as basic enabling technology to improve road efficiency and safety.

An important aspect to take into account is the need of using available low-cost sensors, in order to develop scalable solutions, which can be implemented at large scale and facilitate new driving coordination paradigms [7]. Therefore, the basic sensing technologies must be improved by powerful data processing techniques to handle high-performance expectations, resilient to main causes of faults and lacks of availability/integrity in sensors.

Localization by Global Navigation Satellite System (GNSS) has become a ubiquitous facility in outdoor conditions. This system presents significant variations of quality and reliability depending on the conditions and available enhancements. In urban outdoor conditions the accuracy is typically around 20 m (1 sigma) or more, depending on the number of available satellites and geometrical configuration (dilution of precision, DOP), propagation and especially on the presence of a multipath, a very common situation in urban conditions, whose the worst case is referred as the “urban canyon” problem [9], when the direct path is totally occluded and receivers only make use of signals bounced off walls of close buildings, with the corresponding degradation or even loss of any solution.

There are different enhancements for GNSS, usually classified in Ground/Space Based Augmentation Systems (GBAS and SBAS). The European Geostationary Navigation Overlay Service (EGNOS) is the European reference for SBAS system, with 33 ranging and integrity monitoring stations, while Wide-Area Augmentation System (WAAS) is the Federal Aviation Administration’s reference in the USA. GBA systems consist of ground antennas which transmit differential corrections by VHF data broadcasts to the receiver. An example is the US Local Area Augmentation System (LAAS), used in the proximity of airports to guarantee maximum integrity in Global Positioning System (GPS) position, but this idea is being available in many other environments [10,11].

Besides the ubiquity of GNSS receivers, the recent advances in low-cost inertial sensors based on micro electro-mechanical systems (MEMS) technology [12] has made them emerge as the other big reference technology for navigation. The inertial navigators contain a set of accelerometers in orthogonal axes and aligned gyroscopes which sense vehicle turn rates and accelerations in the body frame. The processor obtains the attitude of vehicle by integrating angular rate measurements in time, and then the position is computed and continuously updated with respect to an initial solution with the projected accelerations measured on body frame.

So, GPS and Inertial Navigation System (INS) sensor systems are complementary key technologies, and a carefully designed sensor fusion process can be used to provide a navigation solution. This type of systems can be explained in simple words as enhancing GNSS with dead-reckoning capability, so that accurate navigation remains available for a certain amount of time when the GNSS signal data becomes unavailable or seriously degraded. However, experience indicates that this solution can be very limited, and the time to support outages or degradation of GPS position is not much longer than some tens of seconds due to very quick drifts in time. GPS/INS fusion is vulnerable to residual errors so a continuous monitoring of the process is necessary to guarantee that the quality of navigation is acceptable, minimizing the effect of these factors during GPS availability drops.

In this article, a modified fusion methodology is explored using adaptive non-linear filters (Unscented Kalman Filter, UKF), which are continuously monitored by a contextual reasoning process, to provide improved performance. The system features a cascaded architecture, separating attitude and kinematic filters, to create a loosely coupled closed-loop scheme that continuously estimates the INS biases to correct them and exploit whenever the GPS data is degraded or unavailable. The system includes explicit knowledge reasoning about vehicle dynamics, to adapt the model to the real conditions. Conditions such as stops, straight motion, lane changes, turns, roundabouts, are considered in the model. Besides, there is a GPS monitoring system with rules depending on conditions based on extra information (availability and age of differential corrections, number of satellites, DOP value, standard deviation, *etc.*). This is applied to weight the fusion parameters or switch the bias estimation processes accordingly to the conditions. Additional external information, such as the presence of blocking buildings and trees creating multipath problems could be considered in a future step, together with the integration of static databases about road and terrain elevation.

The main contribution is the proposal of a robust and adaptable solution, exploiting the good trade-off between non-linear estimation and efficiency of UKF, and including explicit domain knowledge to drive the algorithms.

The proposed system has two features that make this problem more complicated to solve, and are thus key to understand the contribution of the work. The first one is that the sensing system is a simple ensemble of low cost commercial off-the-shelf (COTS) sensors that can be mounted anywhere in the target vehicle. This circumstance increases the difficulty of the problem, since the sensors can be subject to additional dynamic effects that have not been explicitly modeled –such as rolling in corners due to the lateral acceleration.

The second feature is that the sensors have not been calibrated before executing the experiments. Instead, the system automatically corrects those effects online. Continuous self-monitoring and adaptation is necessary to work with the selected low cost sensors, in order to compensate the varying biases and errors over time.

The built system gets good comparison with respect to other recent proposals that assume high quality of sensors and perform an initial calibration of the system. The experimental results, with approximately 50 km in different and representative conditions show the competitive performance of the proposal.

The article is organized in six parts. Section 2 describes the related works, with special emphasis on the architectures and fusion algorithms applied in the domain of car navigation, and other context-aided tracking algorithms. Section 3 presents an analysis of sensor behavior in the operational conditions, to justify the approach detailed in Section 4, which explains the theoretical foundations, architecture and algorithms. The validation and comparative analysis is developed in Section 5, and the conclusions of this study are presented in Section 6.

## 2. Related Works

Latest advances in low-cost sensors, communications and microprocessors are fostering the development of new sensing capabilities for Intelligent Vehicles, allowing these vehicles to aid the driver in maneuvers such as stability control of the vehicle or pedestrian safety in urban environments. The advancements in these mentioned technologies are being extended nowadays to solve complex tasks in the forthcoming Intelligent Transportation Systems, which require normally the combination of sensors and computation to accomplish a reliable solution. The integration of inertial and GNSS data implies the joint estimation of position and orientation, also referred to as the “POSE” estimation problem. Despite the noticeable interest in this problem (UAVs, robotics or aerospace, among other research communities), the proposed solutions are diverse and heterogeneous, without a universally accepted one. The problem requires from deep analysis of field data, with simulation analysis, to customize the algorithms performance accordingly to the application requirements. Attitude estimation appears in the core of the POSE problem, since IMU measurements are referred to the body frame.

Different architectures have been analyzed for sensor fusion in navigation. The uncoupled integration, which uses the output of the navigation solutions from a GPS receiver and an INS to combine independent solutions [13,14], is the simplest solution. However it is hardly useful in practical conditions: in most of the cases the stand-alone INS system presents drift errors increasing with a quadratic dependence on time. Alternatively, in the loosely and tightly-coupled integration, only one centralized estimation filter is used, and the positions measurements (loosely coupled) or pseudoranges measurements (tightly coupled) are directly employed in the filter together with the IMU measurements. These architectures usually include estimation of local sensors errors in the state vector to correct IMU inputs in the aircraft dynamics [15]. Finally, other practical approach, when possible, consists in dividing the estimation problem in several phases. Such approach is the cascaded architecture [16–18]. This approach is appealing from practical aspects, since it allows independent design of separated components in state vector.

The typical approach with respect to estimation algorithms has traditionally involved extended Kalman filters (EKF) to linearize the process and measurement models, usually involving highly nonlinear equations to relate coordinate frame transformations in the measurement model. However, this model relies on linear approximation of a nonlinear system, a complicated mathematical task and sometimes leading to bad performance [19]. In navigation, there are diverse approaches for attitude determination, using both Euler or quaternions to represent orientations [18,20–22]. The issue of how to represent attitude is one of the first decision problems in the fusion system, since all of them violate the Kalman filter’s assumptions of linearity and Gaussianity. Besides this problem with linearization, other factors identified as problematic for Kalman filter in INS/GPS fusion are the dependency on sensor models, INS drift errors, and simplified kinematic models.

With the availability of more computation power, recent works have employed more advanced techniques, like the unscented Kalman filter (UKF) to avoid linearization while providing estimates that capture the statistics of the target distribution more accurately [23,24]. An integrated approach to simultaneous attitudinal and positional estimation is described by van der Merwe and Wan [15], who apply a UKF to estimate a joint Gaussian distribution over orientation and position for an unmanned aerial vehicle (UAV). The resulting filter is found to be more accurate than an EKF used for the same purpose. A constrained unscented Kalman filter algorithm has been proposed in [25] to fuse differential GPS, INS (gyro and accelerometer) and digital map to localize vehicles for ITS applications. The state vector includes accelerometer and gyro biases, and the UKF non-linear character is employed to include some state constraints from the surface geometry.

Other advanced nonlinear filtering algorithms can be used in navigation, e.g., the particle filter related algorithms. Cheng and Crassidis [20] have recently demonstrated how particle filtering may be applied to the problem of spacecraft attitude estimation. Furthermore, [15] shows how this technique can be used for joint estimation of attitude and position in a unified framework. For accurate attitude determination, it is possible to employ extra sensors which provide direct observations, e.g., magnetometers, multi-antenna GPS systems [6], or terrain-aided systems [26]. In the integration of a single GPS receiver antenna and an IMU without redundant attitude information, the INS attitude and sensor bias errors are weakly observable.

Multiple model filters are another extension to estimation. The family of tracking filters called Interacting Multiple Models (IMM) allows the characterization of dynamics behavior according to one of several alternative modes, each mode with an associated probability, and transition rates defined through a discrete Markov process [27]. In [28] an Interacting Multiple Model (IMM) is used. It does not use biases in state vector either, but authors propose different dynamic models and IMM combination: constant velocity (CV), constant acceleration (CA), and constant turn (CT) models. The identification of stops situations is used to activate calibration processes, for instance knowing that during stops, accelerations and rotation rates should be zero.

In [29], an extended Kalman filter is used to integrate data from vehicle sensors consist of wheel speed sensors, steering angle encoder, and a fiber optic gyro. The process model consists of a bicycle-dynamics model, a higher-order model which estimates slip, roll and steering angle. The state vector is simplified to position, velocity and orientation (5D), assuming that optic gyro biases are negligible. With an analogous strategy to facilitate inertial calibration, in [30], GPS, INS and laser scanners are fused in urban environments, proposing the exploitation of complementary nature of GPS and laser scanner sensors in urban conditions (when GPS is occluded by buildings is when laser images become available). A Kalman filter estimates inertial error states using as observables the measurement domain of GPS and laser scanner (range-domain data fusion). The problem of designing complex sensor fusion systems, with accurate modeling, performance adjustment, *etc.*, has been analyzed from the point of view of machine learning, heuristic search and global optimization areas of computational intelligence [31].

In the specific case of navigation and GNSS/INS fusion, some approaches for fault detection of the sensors have been developed. Thus, Noureldin *et al.*[32] proposes dynamic Neural Networks to learn models of INS position and velocity errors before combination with GPS data, allowing adaptation to time-varying errors. The configuration of aircraft navigation sensors was chosen by classifying the probable position accuracy obtained from the various possible sensor combinations. In [13], a GPS/IMU fusion based on contextual variables is proposed. They define fuzzy concepts considering the relative quality of GPS and IMU positions, using predefined thresholds and rules of combination to protect the fusion from degraded data. This paradigm of combining symbolic knowledge (rules) and numerical data to classify sensor quality and avoid corruption of fusion was initially applied with induction algorithms [33], and more recently in [34], based on contextual variables about health of GPS signal and geometry conditions (average fading and fading satellite ratio).

Finally, the use of contextual information and knowledge is a key element in advanced fusion systems, to refine the models for objects and sensor behaviors accordingly to situation. High-level information about the environment, such as surface structure, constraints, expected behavior, *etc.*, may allow the right interpretation of sensor data and the adaptation/optimization in a great deal of fusion system performance. Examples of sources of knowledge, additional to sensor data, are: constraints in motion depending on local topography [35,36], trafficability constraints [37], expected maneuvers motion patterns depending on situation [38], *a priori* known relationships between entities and environment [39,40], *etc.*

In navigation, the use of external contextual information can be a key aspect to improve the robustness and adaptability of systems. There are numerous proposals integrating static data such as topographic terrain models and road networks, while the use of a dynamic representation of contextual situation with reasoning techniques is much more scarce [41,42]. This article presents a proposal to integrate an explicit representation and reasoning of contextual situation in the estimation algorithms, combining the power of advanced non-linear techniques with the flexibility of expert systems to increase adaptability to situations and correct the typical problems with available moderate-cost navigation sensors. A distinguishing contribution with respect to previous approaches is an explicit representation of dynamic situation of car, different from other approaches employing static configuration. This allows adaptability of estimation algorithms to generate accurate solutions, with an on-line calibration process based on the state of car trajectory to correct sensor deviations. An advanced non-linear estimation algorithm (UKF) is continuously adapted considering the contextual situation, increasing the robustness and accuracy of the fused output. This hybrid approach allows the adaptation of fusion algorithm accordingly to the situation, without complex manual adjustments or specific analytical information.

## 3. Sensor and Scenario Analysis

**Figure 1.**
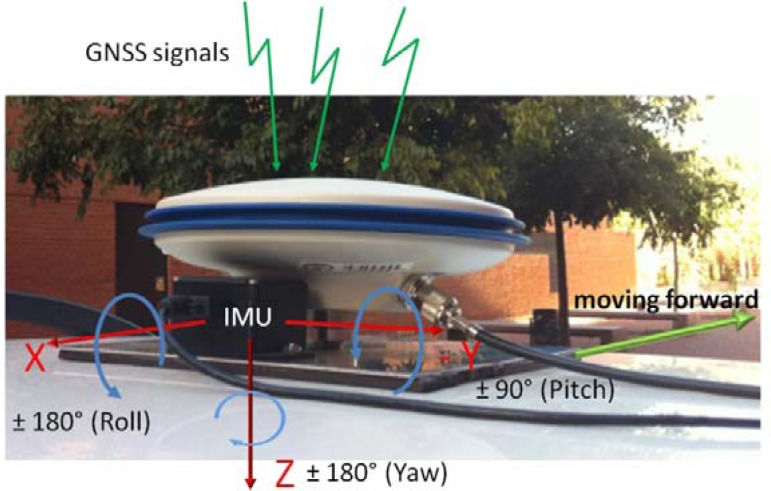
Integration of GNSS antenna of rover receiver and IMU in a platform over the roof of the vehicle.

The device descriptions and system integration are explained in this section. The first sensor is a Differential Global Positioning System (DGPS) which is based on a base station that transmits differential corrections in real-time to a rover station moving in urban environment. The second sensor is an Inertial Measurement Unit (IMU), which integrates three accelerometers and three gyroscopes. The integration of both devices (DGPS + IMU) for sensor fusion is presented in Figure 1. It displays the two sensors on the platform that attaches them to the vehicle roof. The IMU axes are indicated on image with red arrows and their rotation directions (roll, pitch and yaw) are displayed in blue color. This integration allows the study of both devices in good conditions for sensor fusion establishing the same reference through overall experiments.

The GNSS receivers that have been selected for base and rover are two NovAtel OEMV-1G boards, which offer GPS + GLONASS L1 tracking and provide reliable positioning even in obstructed sky conditions. The receivers are embedded on a NovAtel compact enclosure (FlexPak-G2-V1G) for outdoor applications as base station and vehicle position in urban environment.

The rover receiver calculates solutions based on two modes. The first mode is single point position mode (SINGLE mode), which utilizes all available GPS satellites in the position solution without differential corrections. The second mode is differential mode (DGPS mode), where the base station at an accurately known location transmits range corrections to the rover receiver. The update rate associated with SINGLE or DGPS modes is 1 Hz and the rover receiver automatically switches between both modes, where DGPS mode has priority if appropriate corrections are received.

**Figure 2.**
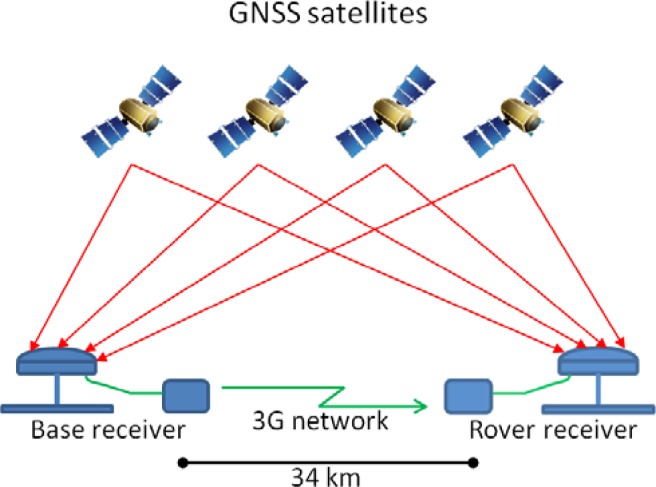
DGPS mode with 34 km baseline and 3G network for sending differential corrections over internet.

The rover receiver uses L1 C/A-code data for differential solution due to advantages in urban environment instead of using carrier-phase DGPS. The carrier-phase DGPS such as Real-Time Kinematic (RTK) has disadvantages for its use in highly dynamic conditions. Then, the experiments of this work are performed with a DGPS system using L1 C/A-code data for differential solution where accuracy is less than 1 meter. Figure 2 shows the architecture of DGPS mode used in this work that is based on 3G network to establish the communication between the base and rover receivers, and allows to send differential corrections from the base to the rover in real-time.

The “optimal conditions” term in SINGLE mode refers to observing six or more healthy satellites and relatively low multi-path (to assure enough quality of the received data). In the case of differential mode, it requires at least four common satellites available both at the base and rover.

The Inertial Measurement Unit (IMU) is a MicroStrain 3DM-GX2, which integrates a triaxial accelerometer, triaxial gyroscope and triaxial magnetometer. IMU data is used to estimate the navigation solution when GPS is not available due to the interruption of satellite signals, which is typical in urban conditions such as driving in a tunnel or within an urban canyon between screening buildings, among other cases.

In Section 5, results will be analyzed from experiments that acquired data from both devices (DGPS + IMU) along 50 km of driving through urban environments and a mountain pass road, both cases with different speeds. We include here some samples illustrating the behavior of the rover GPS device (DGPS or SINGLE mode) in some cases, taking special attention to cases under non-optimal conditions.

**Figure 3.**
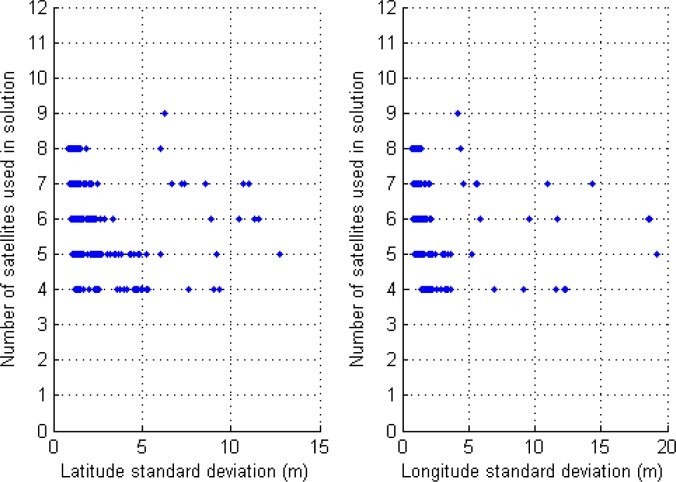
Accuracy of the GPS device *vs.* the number of satellites used in the solution when the rover receiver utilizes DGPS or SINGLE mode.

The accuracy of the GPS device changes if the rover receiver uses DGPS or SINGLE mode. Figure 3 displays the performance of our system based on an experiment with 1,190 GPS data, where high values of standard deviations (x-axis) are caused by short activation time of SINGLE mode, and low values correspond to the rover receiver using differential corrections. Thus, both graphs, as is expected, show in DGPS mode a correlation between the number of satellites used in the solution and the standard deviation; if the number of satellites used in the solution decreases then the latitude and longitude standard deviations increases, as can be observed, when the standard deviation increases towards 5 m in both graphs.

Therefore, the following scenarios delves into the technical details of the GNSS receiver that causes inaccuracy of the position in urban environments and other special cases selected to demonstrate the improvement of sensor fusion.

Some cases of low accuracy caused by switching from DGPS to SINGLE mode are also presented. In our system, DGPS mode loss can be caused by GPS signal unavailability or 3G network outages, and thus the recovery time is variable and a technical detail of the receiver. The age of the corrections in DGPS mode, and its effect in the accuracy of calculated solution has been studied in depth. It constitutes a useful indicator for predicting anomalous behavior, since high values of differential ages cause a decrement of accuracy in DGPS mode. The accuracy in DGPS mode can be also affected by baseline length effect, atmospheric errors, satellite clock and ephemeris errors.

Many researches use only Dilution of Precision (DOP) to characterize the position accuracy, as proposed in [43], which is a numerical representation of satellite geometry. Lower DOP values generally represent better position accuracy, although a lower DOP value does not automatically mean a low position error [44]. Nevertheless, the proposed work uses five parameters, as provided by OEMV-1G NovAtel receiver: the standard deviation of latitude, longitude and height errors; the age of corrections; and the number of satellites used in solution. These five parameters are related to accuracy of position and its reliability, this multi-parameter information is used to tune the sensor quality model employed in the fusion algorithm.

The accuracy of GPS and DGPS positions calculated in this work by the rover receiver mainly depends on the number of satellites that are used in the solution, as well as their geometry, that is quantified by dilution of precision (DOP) parameter. There are other factors that affect also the accuracy of GPS, such as multipath, ionospheric or tropospheric effects. Multipath appears mainly in urban environment where objects reflect GPS signals. This type of error depends on local reflection geometry near each receiver antenna [45].

Some scenarios have been selected from a dataset containing 50 km of data composed of GPS (SINGLE and differential mode) and IMU measurements in real-time, where data acquisition is 1 Hz for positioning system and 50 Hz for inertial sensor. Common factors affecting accuracy in urban environments have been registered together with the measured magnitudes: the standard deviations (latitude, longitude and height), the age of corrections and the number of satellites used in solution.

**Figure 4.**
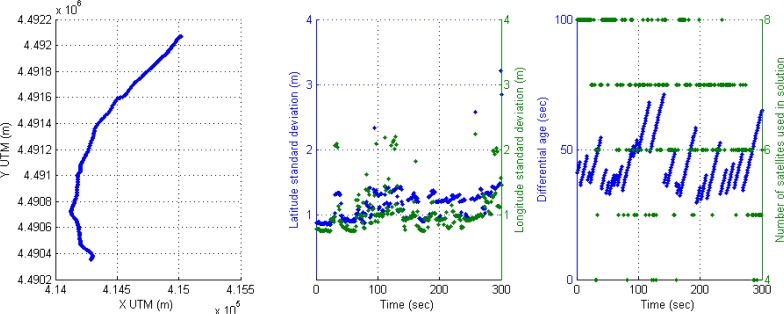
Soft urban environment where differential correction is active: (**a**) Left graph, DGPS trajectory. (**b**) Middle graph, DGPS accuracy. (**c**) Right graph, differential age and number of satellites used in solution.

For instance, the following scenario is selected to show the behavior of the positioning system in an urban trajectory with a high number of satellites used in solution and active differential correction for 300 s, that is, a vehicle trajectory in a soft urban environment. The accuracy is high through the whole trajectory, however it can be observed that DGPS accuracy is reduced when only four satellites are available. This effect of inaccuracy can be seen in Figure 4; the left graph displays DGPS trajectory in Universal Transverse Mercator (UTM) geographic coordinate system, where X and Y coordinates are related to easting and northing coordinate pair. The middle graph shows latitude and longitude accuracy in meters appearing some cases of accuracy reduction around 100 s (2 m and at the end of the graph (2–3 m). The right graph presents the performance of the receiver in DGPS mode where differential ages and number of satellites are according with accuracy, but when four satellites are only used in solution, the DGPS mode loses best conditions and accuracy is reduced.

A typical behavior of the receiver in soft urban environment is the commutation between differential mode and GPS SINGLE mode, causing also the corresponding drop of accuracy. This effect can be noticed if the value when differential age is zero.

**Figure 5.**
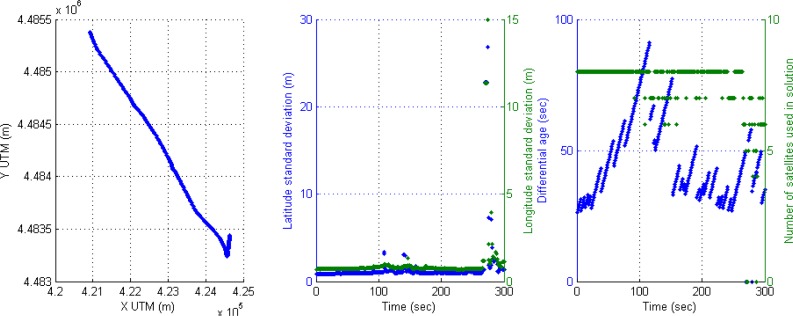
Effect of the high value of the correction age at 110 s and the passing under a motorway at the end of experiment.

Another effect (Figure 5) can be seen around second 110, when a high value of the corrections age appears (83 s) and a reduction from eight to six available satellites, thus the accuracy of positioning is reduced again in differential mode. The second effect in this Figure 5 appears at the end (second 280) where the vehicle is passing under a motorway through a short tunnel, the result in the rover receiver is the loss of differential mode and zero satellites used in solution for three seconds leading to inaccuracy in positioning.

**Figure 6.**
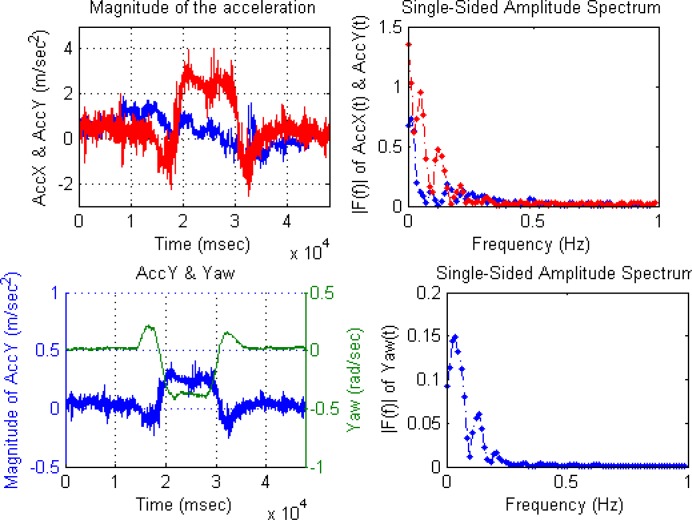
Time-domain and frequency-domain representations of X-axis, Y-axis acceleration and Yaw rotation rate.

With respect to the IMU device, accelerometers and gyroscopes measurements were acquired at 50 Hz for sensor fusion. The following IMU data is an example when the vehicle is performing a 360 degrees roundabout (Figure 6). The first behavior of the vehicle is a deceleration to enter in the roundabout, the next step is a turn of 360 degrees, and finally, the vehicle accelerates at the end of the roundabout. The figure displays the time-domain data acquired while the rover is moving at the roundabout, where upper-left graph is a time-domain representation of X-axis and Y-axis magnitudes of the acceleration (m/s^2^).

The frequency-domain representation allows us to observe some errors in IMU data, as presented in [46], where Fourier Transform (FT) is used to estimate parameters such as, bias, scale factor, and misalignment of the accelerometers and rate gyros. As an example, the bias component can be found at the 0 Hz value of the FT. The upper-right graph of Figure 6 are their frequency-domain representations using the fast Fourier transform (FFT) [47]. Lower-left graph presents again Y-axis magnitude of the acceleration (m/s^2^) to be compared with Yaw rotation rate (rad/s) of Z-axis, and lower-right graph is the frequency-domain representation of Yaw signal. Both amplitude spectrums display only low frequencies (0–1 Hz) to show the 360 degrees roundabout. The amplitude of the discrete frequency component at 0 Hz displays the non-time-varying component of the data.

The X-axis deceleration and subsequent acceleration in the 360° roundabout can be observed in time-domain graph. The roundabout evolution is observed in Y-axis acceleration and Yaw rotation rate time-domain graphs, where both signals show inverse correlation. Frequency-domain of both signals display two discrete frequency components that belong to two short turns right and one long turn left to complete 360° roundabout. Therefore, frequency-domain is useful to estimate bias parameter and effects caused by vehicle dynamic.

## 4. Proposed Solution: Context-Aided Sensor Fusion

### 4.1. Rationale

The architecture used in this work takes as starting point a basic sensor fusion scheme for navigation, similar to others proposed in existing literature. It is extended to integrate the knowledge about the expected dynamics of a ground vehicle in some particular road driving conditions.

Some of the analyzed previous works integrate high-accuracy inertial measurement devices (*i.e.*, Laser Ring Gyroscopes) for solutions with an accumulated distance error below 20 meters after 5 min of dead-reckoning navigation [55]. However, such solution requires a budget around $30,000. The article [48] compares the accuracy of low, medium and high quality sensor sets, using the best one as a ground truth.

Our proposal is more aligned with the work presented in [49]. It uses an UKF to fuse the output of an electro-mechanical IMU, digital compass and GPS sensors. The GPS system used in those experiments has a lower precision that the one on this work, but they include the output of a digital compass which provide an absolute reference for car attitude. In spite that the obtained results are not detailed, and no explicit performance metrics are provided, they conclude that the accuracy of the system is adequate.

In the two works presented above, authors boldly emphasize the need to perform an initial alignment and calibration of the sensors. The novel part of our proposal is that, apart from using a low/medium budget system, it is able to automatically perform calibration online. The implemented system exploits explicit domain knowledge to detect favorable situations where this task is possible. This is a key factor for building commercial off-the-shelf systems, where some performance can be sacrificed in favor of a reduced cost and simplified deployment.

### 4.2. Architecture

**Figure 7.**
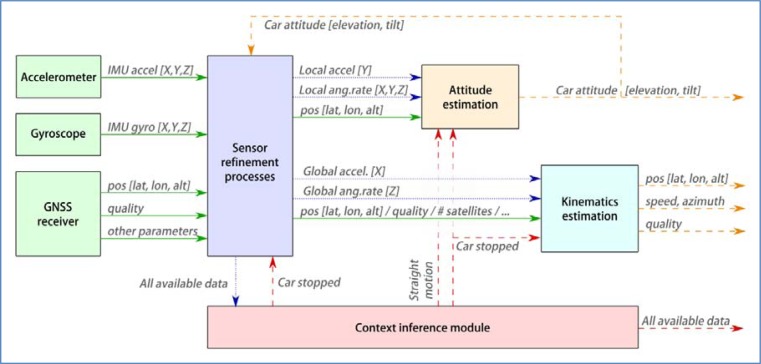
System architecture.

Figure 7 shows the architecture of the system. In general, information flows from left (sensors) to right (output of estimation processes). The main exception resides in the module that infers contextual information, whose products are accessible to every other component in the system. It can also be seen that the result of attitude estimation is fed back into the sensor refinement process.

The figure shows how data moves around the system. Solid lines represent raw data as captured by the sensors, blue dotted lines refers to refined sensor information (original data that has been processed to compensate known errors, or to produce features that summarize a number of raw values). Finally, dashed lines are reserved for new data that has been obtained by advanced processing techniques such as filters or reasoning algorithms. The meaning of each data component will be detailed in the corresponding section, where it is produced.

#### 4.2.1. Notation and Conventions

Let us define, beforehand, some conventions and rules about notation followed over the rest of the document. The coordinate reference systems used by the sensor fusion solution are Cartesian, right-handed. World coordinates follow ENU convention, with its origin located near Colmenarejo Campus of the University Carlos III de Madrid (Madrid, Spain). All the tests took place within a distance of 20 km. The three axes of world coordinate system will be referenced as {*X*, *Y*, *Z*}.

Both the IMU, GPS and vehicle are considered to share a common reference system, that will be named as “Local”. Its axes are named {*X^L^*, *Y^L^*, *Z^L^*}, with *X^L^* following the motion direction of the car (positive when the car moves forward, negative when reverse), *Y^L^* growing towards the left side of the vehicle, and *Z^L^* pointing upwards. For simplicity, the origin matches that one of the IMU. Raw GPS, accelerometer and gyroscope measures are transformed to this reference system before being used by the system.

The word “attitude” is referred to the vehicle/IMU. It express the orientation difference (rotation) between the local axes {*X^L^*, *Y^L^*, *Z^L^*} and world reference system {*X*, *Y*, *Z*}. This works represent rotations either as matrices or as global Euler terns *Eul_G_*{*x*, *y*, *z*}. The latter express the attitude as a sequence of three rotations around global axes {*X*, *Y*, *Z*}. These individual rotations are named tilt, elevation and azimuth, respectively.

Gyroscope readings express angular rates around local axes {*X^L^*, *Y^L^*, *Z^L^*}. They will be also referred as roll, pitch and yaw, respectively.

### 4.3. Inference of Context Information

The proposed architecture defines a context inference module, in charge of inferring useful information about the vehicle and its surroundings: the *context*. The decision of configuring it as a transversal module is not casual, it has a clear motivation.

The use of contextual information to enhance performance is not new. However, and in spite that the use of this kind of information has been proved to be useful and valuable, it is usually applied in very specific *ad hoc* processes, without an explicit architecture to represent and exploit this knowledge.

Our proposal aims to spread contextual knowledge as if it was provided by another sensor, as proposed in [50]. It should be usable by the whole set of processes, and, thus, it must be accessible to every other component in the system. The design requirements for this module are:

- It must provide up-to-date online information. This means that it must work only over past and present sensed information, *i.e.*, no need of future data.
- The results must be readily available for any component requesting them. The result of such request will be a self-contained data element, such as a tuple of values with an associated timestamp.

In order to avoid risks related with “data incest” or “double counting”, *i.e.*, combining information which is correlated after being affected by common data sources, interdependences have been minimized by using the information as closest to the raw sensor readings as possible.

**Figure 8.**
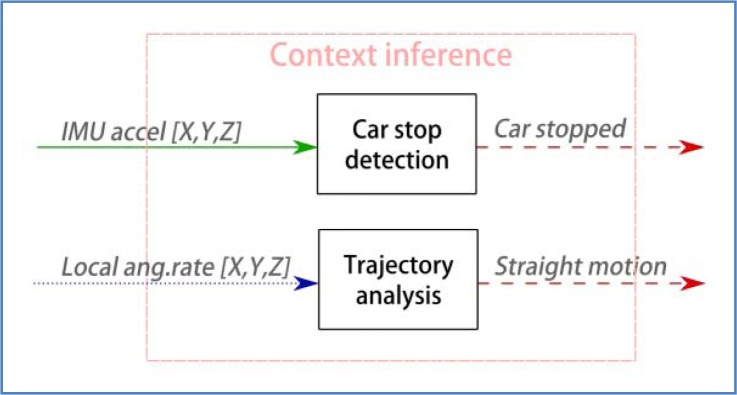
Context inference module. Current implementation.

For this work, context inference has a limited reach (Figure 8). The implementation exploits a trajectory analysis module that detects two specific situations: when the vehicle is stopped, and when it is moving in a straight road lane. This simple approach has allowed a significant improvement in the dynamic correction of sensor drifts and estimation of vehicle pose. The next paragraphs explain the implementation of these components.

#### 4.3.1. Detect Car Stops

The selected stop detection algorithm is solely based in IMU readings to improve the availability of the service, since GPS is subject to outages. Although using the car acceleration values for this task could seem the most suitable approach, it is important to notice that this sensor is subject to several effects that can cause some algorithms to fail. First, accelerometer measures can be biased, and those biases are usually unknown to us: our proposal tries to correct those effects online. In second place part of the gravity acceleration is usually transmitted to *X^L^* and/or *Y^L^* axes of the accelerometer.

**Figure 9.**
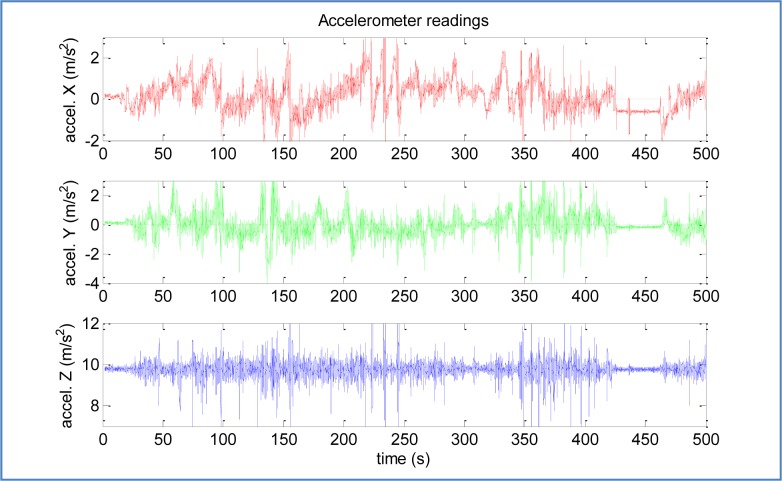
Sample accelerometer readings featuring two stops around t = [0;15] and t = [430;460] seconds. The varying bias during stops makes the raw signal not adequate for detecting stops.

Both problems together make very difficult (if not impossible) to estimate if the acceleration of the car is zero at some point. This can be seen in Figure 9, especially in axis *X^L^* readings, where the acceleration registered in the stops differs in 0.9 *m*/*s*^2^ because in the second stop the car is on a steep road (approx. 5 degrees).

However, a significant amount of vibrations are transmitted to the IMU while the vehicle is in motion, even over the smoothest terrains. The three axes show a near constant reading during the time spans where the car is not moving.

Thus, this sub-module uses the amplitude of accelerometer measures in a window of time (maximum measured value minus minimum) as an indicator of a “flat” reading. The advantages of the proposed method include:

- Simple and fast computation (maximum/minimum of a reduced amount of numbers)
- Independency of biases, attitude and other conditions

However, it requires setting a suitable threshold for determining when the car is stopped (0.5 m/s^2^ in our case). Although this can be easily set taking into account the expected random error according to the manufacturer, more robust alternatives can incorporate GPS position estimates to enhance the stop detection.

#### 4.3.2. Trajectory Analysis

Our system features a robust algorithm for detecting certain trajectory features that have a special meaning. In this work, the implementation has been limited to detecting where the car is driving over a lane in a straight fragment of the road, with no lateral or longitudinal maneuvers. As well as it happened with car stops, this information can be useful for additional fusion or sensor refinement processes, although it can be applied to more advanced reasoning processes.

Turns are clearly indicated by the gyroscope yaw reading (around *Z^L^* axis), tightly related with changes in the azimuth of the vehicle. Detection of straight movement takes the average gyro reading in a window of time (typical values range from 0.5 to 2.0 s), and determines a straight movement when the absolute value is under a threshold.

This detection requires, however, a non-biased gyroscope reading which is provided by the sensor refinement module. The details are provided in next section.

### 4.4. Sensor Refinement

Sensors are subject to external and internal conditions which affect their performance in different ways. Sometimes, the sensor itself will be capable of providing a self-assessment of its observations quality, as in the case of GPS measures that include the number of available satellites or the precision of the calculated solution.

For some others, however, it is necessary to apply external checks for detecting degraded performance or faulty sensor conditions. As an example, the bias or systematic deviation of micro-electronic based accelerometer and gyroscopes is stable at short/medium term, but suffers a slow drift related with factors such as the internal temperature of the device.

This means that an initial calibration is not enough for keeping a system running over long periods of time. The best solution involves monitoring the quality of sensor readings, and calculating the parameters that correct them when possible.

**Figure 10.**
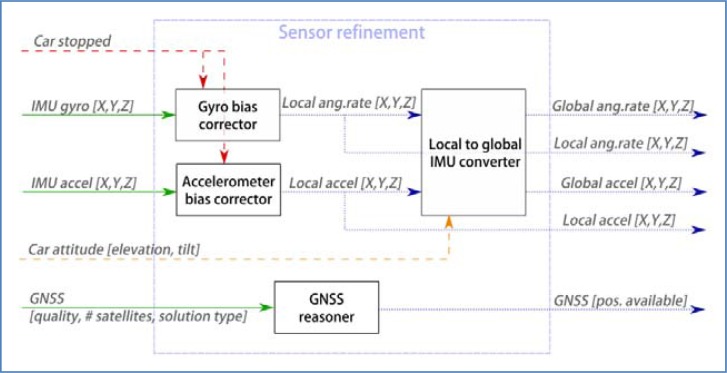
Sensor refinement module.

In the proposed system, sensor refinement is understood as a layer between the sensors and any other process accessing their data (Figure 10). Some of the processes depend on information that is only available in later components, in particular:

- Transforming local IMU readings to world frame of reference requires an estimation of car attitude
- The algorithm for correcting gyroscope bias is active when the car is not moving

This can be solved by introducing a feedback from latter layers. It must be checked that there are no cyclic dependencies or that even under them the system will converge to a stable solution.

Gyroscope bias can be corrected when the car is stopped: the reading on each axis is its bias, plus a random perturbation which can be modeled as Gaussian noise. Therefore, bias is estimated as the average of the readings on each axis over the period where the car is stopped (the reduction in noise variance will be inversely proportional to the number of samples). So, the key to update biases in gyroscopes will be the good inference of contextual information, triggering the process each time the car is stopped.

The case of accelerometer bias is much more complicated than the gyroscope, due to the effect of gravity. With the vehicle stopped, the reading on the accelerometer axes is:

(1) $${\mathrm{acc}}_{{\left\{x,y,z\right\}}^{L}}=b_{{\left\{x,y,z\right\}}^{L}}+R^{T}\cdot \left(g\cdot \vec{z}\right)$$

where *b*_{*x,y,z*}^*L*^_ are the biases of the three local accelerometer axes, *g* is the magnitude of the acceleration due to gravity, *z⃗* is the unit vector which follows the direction of *Z* axis in world coordinates and *R* is a rotation matrix that express the attitude of the IMU (*R^T^* transforms from world-referenced to local-referenced coordinates).

According to the equation above, bias and rotation have to be determined simultaneously. Solving this problem with no prior calibration represents a challenge, since degree-level errors while determining the attitude can be compensated by drifting the estimated accelerometer bias in a reasonable quantity.

Bias estimation is restricted in our model to the *X^L^* axis of the accelerometer, since this reading is the one used in the kinematic model of the vehicle. It is possible to see that, if car attitude *R* is expressed as global Euler tern (tuple of 3 elements), then azimuth angle can be safely ignored. Let us parameterize the attitude of the IMU using only tilt and elevation angles, *R* → *R_ψ,ϕ_*

Applying the same reasoning process followed in reference [29], instant accelerometer reading for X axis when the vehicle is stopped can be written as *acc_X_* = *b_a_*(*X*) + *g* · sin(*ϕ*), which leads to (2):

(2) $$b_{a}\left(X\right)={\mathrm{acc}}_{X}-g\cdot \text{sin}\left(\phi \right)$$

### 4.5. Vehicle State Estimation

There are any navigation-related works that describe the vehicle using a 2D model, as in [29], where GPS is combined with a wheel speed detector and a gyroscope. This simplification works well in the case that it is not needed to correct acceleration measures, and the error of using gyroscope readings in their own frame of reference does not introduce significant errors due to the features of vehicle dynamics.

However, this simplification cannot be applied in many car driving conditions. The best example is probably that one related with accelerometer readings and the effect gravity has on them: a vehicle driving uphill at constant speed in a *β* degrees slope (elevation angle) registers a residual acceleration along its local *X^L^* axis with magnitude *acc_Rx_* = *g* · sin(*β*), that is approximately $0.17\frac{m}{s^{2}}\cdot \deg$ for small angles *β* < 10 *deg*. This residual acceleration is large enough to introduce important errors when the system has to predict vehicle state using only inertial measures, even for short periods. The same can be applied to accelerations along *Y^L^* axis depending on the tilt of the vehicle, and to how gyroscope registers turns depending on road features as the cant.

These arguments would be enough to justify a full state estimation, which can account for the whole possibility of movements that a car can perform in the 3D space. The most straightforward approach consists in applying a single filter that works with a constrained six Degrees of Freedom (6DoF) system. This solution can be found in some of the already cited works, as [25,49].

A two-stage solution has been preferred instead: the first block estimates the attitude of the vehicle for correcting the inertial inputs, and the second one predicts its kinematics using a simpler 2D model that take into account the motion constraints of a ground vehicle. It has been shown that uncoupled solutions offer a poorer performance when compared with loosely- and tightly-coupled formulations [48]. The reason that impelled us to select the uncoupled solution is that it makes easier to apply the implemented sensor refinement and context-based techniques. The next sections describe the prediction and measurement models of the two state estimators, which will be used in two uncoupled UKFs.

#### 4.5.1. State estimation with Unscented Kalman Filter

The UKF is a member of the Kalman family. As the basic Kalman filter [51], it is a recursive algorithm that estimates the state *x̂_k_* of discrete-time dynamic system composed by a mix of partially observable and hidden variables. The estimation is described as a multivariate Gaussian distribution with mean *x_k_* and covariance *P_k_*.

These filters use a mathematical description of how the system evolves over time, the *prediction model f*(·):

(3) $${\hat{x}}_{k+1}=f\left({\hat{x}}_{k},u_{k},v_{k}\right)$$

where *u_k_* is an input that complements the prediction model but does not provide any information about the state by itself, and *v_k_*∼*N*(0; *R_v_*) represents a process noise distributed as a Gaussian with mean zero and covariance matrix *R_v_*.

A series of measurements are received over time:

(4) $${\hat{y}}_{k}=h\left({\hat{x}}_{k},w_{k}\right)$$

which are observations of the true state transformed by a known measurement model *h*(·) and perturbed by a random sample of the observation noise *w_k_*∼*N*(0; *R_w_*) with the same restrictions applied to process noise. The information provided by such observations is integrated into the state estimation during the update step.

The KF is limited to linear prediction and observation models, but it provides a formulation to first predict the probability distribution of the state in a future time instant, and then use a measurement to correct the prediction and reduce the uncertainty:

(5) $$\left\{x_{k},P_{k}\right\}\overset{\mathrm{predict}\left(\Delta t\right)}{\to }\left\{x_{k+1}^{-},P_{k+1}^{-}\right\}\overset{\mathrm{update}\left(y_{k+1}\right)}{\to }\left\{x_{k+1}^{+},P_{k+1}^{+}\right\}$$

The UKF [52,53] is an extension of the original algorithm that allows using nonlinear models. Given a *L*-dimensional state, this filter uses a set of 2*L* + 1 weighted sample points *χ* called sigma points chosen according to the mean *x_k_* and covariance *P_k_* of the state estimation:

(6) $$\chi_{0}=x_{k}$$

(7) $$\chi_{i}=x_{k}+{\left(\sqrt{\left(L+\lambda \right)\cdot P_{k}}\right)}_{i},i=1,\ldots ,L$$

(8) $$\chi_{i}=x_{k}-{\left(\sqrt{\left(L+\lambda \right)\cdot P_{k}}\right)}_{i},i=L+1,\ldots ,2L$$

where *λ* = *α*^2^(*L* + *κ*) − *L* is a scaling parameter, and constants *α*, *κ* are the spreading of the sigma points around the mean and a secondary scaling parameter respectively. $\sqrt{\left(L+\lambda \right)\cdot P_{k}}$ is a matrix square root, and (·)*_i_* represents its *i*-th column.

These points are propagated using the prediction function $\chi_{k+1}^{-}=f\left(\chi_{k},u_{k}\right)$. The new state probability distribution $\left\{x_{k+1}^{-},P_{k+1}^{-}\right\}$ are calculated as the weighted mean and covariance of the sigma points:

(9) $$x_{k+1}^{-}=\sum_{i=0.2L}W_{i}^{\left(m\right)}\cdot \chi_{i}^{-}$$

(10) $$P_{k+1}^{-}=\sum_{i=0.2L}W_{i}^{\left(c\right)}\cdot \left(\chi_{i}^{-}-x_{k+1}^{-}\right){\left(\chi_{i}^{-}-x_{k+1}^{-}\right)}^{T}$$

where the weights for the mean $W_{i}^{\left(m\right)}$ and covariance $W_{i}^{\left(c\right)}$ are given by:

(11) $$W_{0}^{\left(m\right)}=\lambda /\left(L+\lambda \right)$$

(12) $$W_{0}^{\left(c\right)}=\lambda /\left(L+\lambda \right)+\left(1-\alpha^{2}+\beta \right)$$

(13) $$W_{i}^{\left(m\right)}=W_{i}^{\left(c\right)}=\frac{1}{2\left(L+\lambda \right)},i=1,\ldots ,2L$$

Being *β* a parameter that controls the shape of the distribution (*β* = 2 optimal for Gaussian distributions). For extended details on the basics of Kalman-like filters in general and UKF in particular, the excellent references [51,53] are recommended. Next, the UKF algorithm has been particularized to the problems of attitude and car kinematic trajectory, using the cascaded architecture introduced in Section 4.2. In the following subsections, the particular state vectors and non-linear dynamics of each subproblem are presented.

#### 4.5.2. Attitude Estimation Model

Let us describe the attitude of the vehicle as a global Euler tern *Att* = [*ψ*, *ϕ*, *θ*], where *ψ* is tilt, *ϕ* is elevation and *θ* is azimuth. This tern represents a sequence of ordered rotations around the axes of world system of reference. First around X, then Y, last Z.

**Figure 11.**
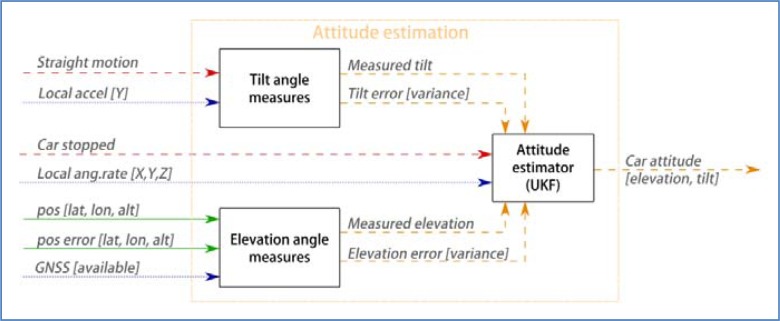
Attitude estimation process.

This part of the system, shown in Figure 11, estimates the tilt and elevation of the vehicle. These two components contain all the necessary information for:

- Subtracting the effect of gravity from accelerometer readings,
- Translate local gyroscope readings to world system of reference. This is of great importance during turns where the car is tilted.

So, the state vector is *x_att_* = [*ψ*, *ϕ*]. Given the gyroscope readings *u_gyr_* = [*g_x_*, *g_y_*, *g_z_*], which represent the angular rates (in radians) around the local {*X^L^*, *Y^L^*, *Z^L^*} axes, the prediction model for estimating the new attitude of the car after a time span *t* follows the procedure described below. First we detail the prediction model, which takes as control inputs the gyroscope readings and carries out a numerical approximation, and then the observation model, which needs external information to infer observations of these magnitudes. The estimates cast by both models are then integrated with the UKF estimation process.

Since gyroscope readings represent a simultaneous rotation around the three local axes at the marked angular rates. That means that the local reference system changes continuously over time. For infinitesimal time increments, the simultaneous rotation is similar to applying three sequential infinitesimal rotations around each one of the axes, with independence of the order. Using matrix form, this can be expressed as:

(14) $$\begin{array}{c} M_{\delta }=M\left(X,\delta g_{x}\right)\cdot M\left(Y,\delta g_{y}\right)\cdot M\left(Z,\delta g_{z}\right)=\\ \left(\begin{array}{ccc} 1 & 0 & 0\\ 0 & \text{cos}\left(\delta g_{x}\right) & -\text{sin}\left(\delta g_{x}\right)\\ 0 & \text{sin}\left(\delta g_{x}\right) & \text{cos}\left(\delta g_{x}\right) \end{array}\right)\cdot \left(\begin{array}{ccc} \text{cos}\left(\delta g_{y}\right) & 0 & \text{sin}\left(\delta g_{y}\right)\\ 0 & 1 & 0\\ -\text{sin}\left(\delta g_{y}\right) & 0 & \text{cos}\left(\delta g_{y}\right) \end{array}\right)\cdot \left(\begin{array}{ccc} \text{cos}\left(\delta g_{z}\right) & -\text{sin}\left(\delta g_{z}\right) & 0\\ \text{sin}\left(\delta g_{z}\right) & \text{cos}\left(\delta g_{z}\right) & 0\\ 0 & 0 & 1 \end{array}\right) \end{array}$$

where *δg_k_* represents the infinitesimal angle rotated after sustaining the *g_k_* angular rate around axis *K* during the infinitesimal time *δt*. By integrating the differential rotation *M_δ_* over time, the total pose change can be obtained.

Our model performs a numerical approximation of this approach. First, the prediction time span *t* is divided in *n* steps of duration $d=\frac{t}{n}$ seconds. The pose change in any of the steps is calculated as a sequential rotation of *u_gyr_* · *d* = [*g_x_* · *d*, *g_y_* · *d*, *g_z_* · *d*] radians around the three axes, which results in the differential rotation matrix *M_d_*. The total pose change after prediction time span is the *n*-th power of the differential rotation matrix, $M_{\mathrm{rot}}=M_{d}^{n}$.

As a side note, our choice for *n* is such that the duration of the step is smaller than *d* = 10^−4^ seconds. The obtained results were compared with those yielded by the widely accepted quaternion kinematics equation, resulting in errors around one part per billion.

The new vehicle attitude can be calculated as:

(15) $$M{\left(x\right)}_{+t}=M\left(x\right)\cdot M_{\mathrm{rot}}$$

Where *M*(*x*) depends on the reduced vehicle attitude *x_att_* = [*ψ*, *ϕ*] expressed as a rotation matrix. Note that pose change matrix post-multiplies the attitude because the rotations are expressed around global axes. Transforming the resulting matrix *M*(*x*)_+*t*_ to Euler notation (again, around global axes) and discarding the azimuth values gives the new vehicle attitude *x*_*att*(+*t*)_ = [*ψ*′, *ϕ*′].

Finally, UKF equations are applied to combine the prediction with asynchronous measures and provide the estimated tilt and elevation angles. Table 1 shows the full attitude estimation filtering algorithm.

##### Generation of Tilt Angle Measures

The tilt angle of the car can be calculated based on the gravity transmitted to accelerometer *Y^L^* axis. As previously stated, the reading on this *Y^L^* axis at a given time is:

(16) $${\mathrm{acc}}_{y}=a_{y}^{L}+b_{y}^{L}+g_{y}^{L}+n_{y}$$

where:

1. $a_{y}^{L}$ is the real lateral acceleration associated to vehicle motion;
2. $b_{y}^{L}$ is the bias of the accelerometer on its Y axis;
3. $g_{y}^{L}$ is the effect of gravity in the local Y axis of the accelerometer;
4. *n_y_* is a random sample distributed as white noise with variance *σ*_*acc_y_*_.

During fragments where the vehicle is moving in a straight piece of road, the car will not be subject to lateral accelerations and it is valid to assume that $a_{y}^{L}=0$. Regarding the random noise, it can be cancelled by averaging several measures representing the same effective acceleration. This also happens during straight motion. If bias has been corrected, we found that in these fragments of trajectory ${\mathrm{acc}}_{y}=g_{y}^{L}$.

The effect of gravity can be calculated by transforming it to local axes. Assuming that the attitude of the car is *x_att_* = [*ψ*, *ϕ*], the rotation matrix that performs such transformation is:

(17) $$\begin{array}{c} M\left(X,\psi \right)\cdot M\left(Y,\theta \right)=\left(\begin{array}{ccc} 1 & 0 & 0\\ 0 & \text{cos}\left(\psi \right) & -\text{sin}\left(\psi \right)\\ 0 & \text{sin}\left(\psi \right) & \text{cos}\left(\psi \right) \end{array}\right)\cdot \left(\begin{array}{ccc} \text{cos}\left(\theta \right) & 0 & \text{sin}\left(\theta \right)\\ 0 & 1 & 0\\ -\text{sin}\left(\theta \right) & 0 & \text{cos}\left(\theta \right) \end{array}\right)=\\ =\left(\begin{array}{ccc} \text{cos}\left(\theta \right) & 0 & \text{sin}\left(\theta \right)\\ \text{sin}\left(\psi \right)\text{sin}\left(\theta \right) & \text{cos}\left(\psi \right) & -\text{sin}\left(\psi \right)\text{cos}\left(\theta \right)\\ -\text{cos}\left(\psi \right)\text{sin}\left(\theta \right) & \text{sin}\left(\psi \right) & \text{cos}\left(\psi \right)\text{cos}\left(\theta \right) \end{array}\right) \end{array}$$

This matrix has to multiply the global-referenced gravity vector [0, 0, *g*]*^T^*. The Y component of the transformed gravity vector will be − sin(*ψ*) cos(*θ*) · *g*. The tilt and elevation angles of a vehicle in normal road conditions are usually in the range [−5°;5°], and hardly ever exceed 10°. This makes possible to apply the approximation cos(*θ*)∼1.

Back to the reading under straight movement conditions, we have that:

(18) $${\mathrm{acc}}_{y}=g_{y}^{L}=-g\cdot \text{sin}\left(\psi \right)$$

The tilt angle *ψ* can be calculated as $\text{arcsin}\left(-\frac{{\mathrm{acc}}_{y}}{g}\right)$. Thus, true tilt angle at time step *t* can be estimated using the average accelerometer reading over a window of *k* samples taken during straight motion as:

(19) $$\psi =\arcsin\left(-\frac{\sum_{i=0..k-1}{\mathrm{acc}}_{y}\left(t-i\right)}{g\cdot k}\right)$$

##### Generation of Elevation Angle Measures

**Figure 12.**
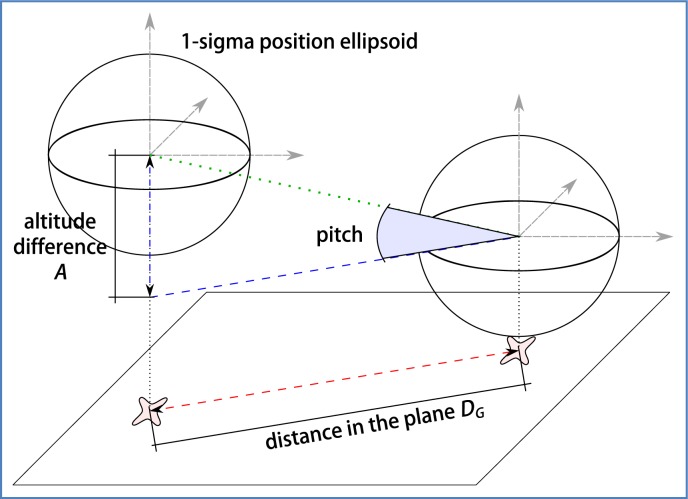
Calculation of elevation angle from two GPS measures.

Raw elevation angle can be estimated using GPS information of consecutive measures, as illustrated in Figure 12. Assuming that GPS measures have been transformed to a Cartesian system of reference, we can calculate:

(20) $$D_{G}=\sqrt{{\left({\mathrm{lat}}_{2}-{\mathrm{lat}}_{1}\right)}^{2}+{\left({\mathrm{lon}}_{2}-{\mathrm{lon}}_{1}\right)}^{2}}$$

(21) $$A={\mathrm{alt}}_{2}-{\mathrm{alt}}_{1}$$

(22) $$\Delta \theta =\text{atan}\left(A/D_{G}\right)$$

This estimation of pitch angle is quite sensitive to the measurement conditions. In one hand, it is important to use two GPS measures close enough in time so that the path of the vehicle between them can be well approximated by a straight line, and also that the elevation angle has remained near constant. On the other hand, the 3D points must be as separated as much as possible so that the error of GPS does not have a large impact on the calculated elevation. Our GPS device provides measures at a fixed rate of 1 Hz. The distance between consecutive positions will depend on the speed of the vehicle.

#### 4.5.3. Estimation of 2D Vehicle Kinematics

For locating the vehicle on a surface, a 2-dimensional model is proposed. We assume no wheel slippage. Let the state vector be *x* = [*p*(*x*), *p*(*y*), *v*, *ϕ*]*^T^*, where *p*(*x*), *p*(*y*) describe the position of the vehicle in the X-Y plane of world coordinates, *v* is the linear speed of the vehicle and *ϕ* is the azimuth angle which marks its course.

The azimuth complements the output of the attitude estimation model, to form the complete attitude vector of the vehicle. The prediction function takes the state of the system, and a control input *u* = [*a_x_*, *ω_z_*]*^T^* formed by the corrected (world coordinates, non-biased) longitudinal acceleration of the car and yaw angular rate.

#### Model for Low Angular Rates

When the movement of the car is near-straight, its kinematic is calculated using the following simple model:

(23) $$x_{t+\Delta t}=f\left(x_{t},\Delta t,u_{t}\right)=\left[\begin{array}{c} p{\left(x\right)}_{t}+v_{t}\cdot \text{cos}\left(\theta_{t}\right)\cdot \Delta t\\ p\left(y_{t}\right)+v_{t}\cdot \text{sin}\left(\theta_{t}\right)\cdot \Delta t\\ v_{t}+a_{x}\cdot \Delta t\\ \theta_{t}+\omega_{z}\cdot \Delta t \end{array}\right]$$

##### Model for Turns

During turns, the prediction function switches to an adaptation of Ackermann steering model [54] for four wheeled vehicles where the two frontal can turn. According to this model, a vehicle with its wheels turned a fixed angle will describe a circle. The radius of this circle can be calculated as the quotient between the linear speed of the vehicle and its angular rate $R=\frac{v}{\omega_{z}}$. This radius is the criterion for selecting between models. The model for turns described here is selected when *R* < 100 *m*, because the expected errors of the simple model will be exceeded by those of the sensors and other estimation processes.

As both position and kinematic data are referred to the location of GPS/IMU sensors, we consider the radius of the turning as the distance between the center of the rotation and the IMU. The origin for the rotation can be calculated as:

(24) $$P_{\mathrm{rot}}={\left[p\left(x\right),p\left(y\right)\right]}^{T}-\vec{x}\cdot L+\vec{y}\cdot R$$

where *L* is the distance between the IMU and the center of the rear axis (*L* ≅ 1.3 *m* in test vehicle), *R* is the radius of the described circle, and *x⃗*, *y⃗* are unit vectors following the direction of IMU local axes. These vectors can be expressed in world-reference coordinates using vehicle azimuth angle, *x⃗* = [cos(*θ*), sin(*θ*)], *y⃗* = [−sin(*θ*), cos(*θ*)].The new location of the vehicle is the result of rotating the old one *ω* ·Δ*t* radians around *P_rot_*, this is:

(25) $$P_{\mathrm{loc}}=\left[\begin{array}{l} p{\left(x\right)}_{t}\\ p{\left(y\right)}_{t} \end{array}\right]-P_{\mathrm{rot}}$$

(26) $$\left[\begin{array}{c} p{\left(x\right)}_{t+\Delta t}\\ p{\left(y\right)}_{t+\Delta t} \end{array}\right]=\left(\left[\begin{array}{cc} \text{cos}\left(\omega_{z}\cdot \Delta t\right) & -\text{sin}\left(\omega_{z}\cdot \Delta t\right)\\ \text{sin}\left(\omega_{z}\cdot \Delta t\right) & \text{cos}\left(\omega_{z}\cdot \Delta t\right) \end{array}\right]\cdot P_{\mathrm{loc}}\right)+P_{\mathrm{rot}}$$

where the old position [*p*(*x*)*_t_*, *p*(*y*)*_t_*]*^T^* is then first translated so that the origin of the rotation *P_rot_* is located at [0,0]*^T^*, then a rotation matrix is applied, and the result is translated back to world-referenced coordinates.

Speed and attitude are calculated as in the first formulation:

(27) $$v_{t+\Delta t}=v_{t}+a_{x}\cdot \Delta t$$

(28) $$\theta_{t+\Delta t}=\theta_{t}+\omega_{z}\cdot \Delta t$$

## 5. Experimental Validation

The experimental validation has been carried out in a set of representative scenarios to show the reliability of the proposed system. In the first place, we present some results about contextual analysis and sensor correction subsystems. The other results display the performance of the filters when GNSS signals are unavailable or severely degraded in complex urban environments.

### 5.1. Evaluation of Context Reasoning and Sensor Correction Subsystems

**Figure 13.**
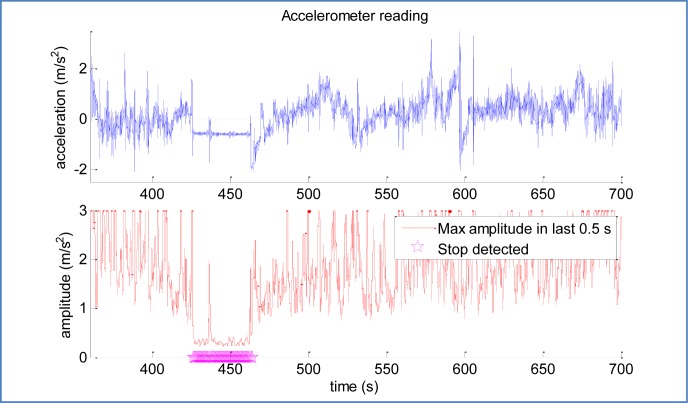
Sample accelerometer readings, processed signal, and output of the car stop detection module. This figure shows the validity of the applied strategy.

The stop detection algorithm is based in measuring the “roughness” of accelerometer output over time. For this purpose, a window of 0.5 s proved to offer good results without introducing a significant delay. Moreover, stops are useful when extend over a few seconds, so that the delay is not usually important. Figure 13 shows the performance of the selected algorithm over the second stop of previous trajectory, demonstrating its validity even with biased inputs.

**Figure 14.**
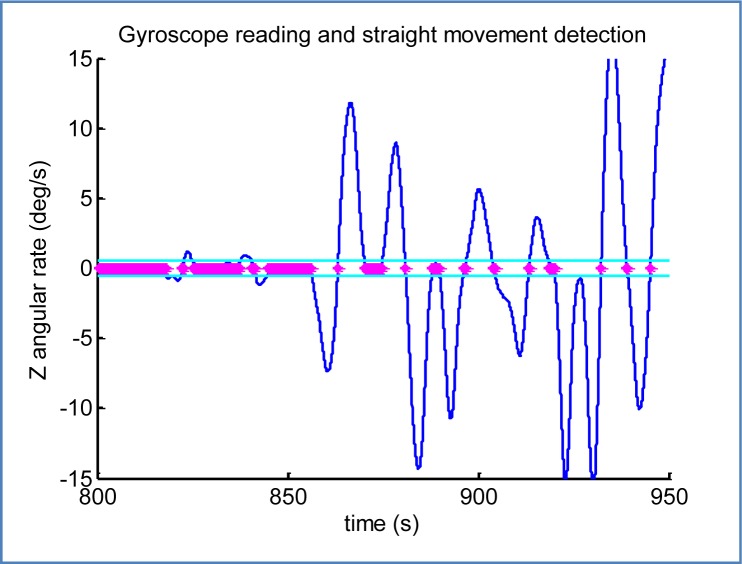
Output of the trajectory analysis module: straight movement detection using accelerometer readings.

For the trajectory analysis part, the non-biased gyroscope reading around *Z* global axis is used. This data element is provided by the sensor refinement module. In Figure 14 is possible to see the raw output of the thresholding criterion that determines when the car is travelling straight, this is, not turning. The selected limit of ±0.5 degrees per second does not completely guarantee a straight movement, but it rather indicates that the readings of the other sensors will not be affected by some of the effects of curves, e.g., car inclination, lateral accelerations.. A further refinement has been implemented by interval analysis to discard fragments shorter than a few seconds, to avoid those detections between linked turns.

It is interesting to see the two small interruptions of the straight movement around t = [820;825] and t = [840;845]; they represent two consecutive changes of lane, the first one to the right and the second back to the left. Readings from t = 860 in advance are part of a curvy mountain road with brief straight segments, revealing a satisfactory performance even with strong slopes.

With respect to bias drift, in this type of sensors is known to be caused by temperature changes, and thus is a slow process. The 15 stops detected in the experiments returned a quite stable estimation of *b_g_* = [0.29, −0.31, 1.05] *deg*/*s*, with a variance *var*(*b_g_*) < [0.01, 0.01, 0.02]*deg*/*s*. This can be explained because all the records were taken in the same day, starting half an hour after the device was mounted and exposed to direct sun light and having reached a stable temperature.

The use of dynamic adaption would have kept the gyroscope calibrated under any other conditions. Taking into account these considerations, two indicators of algorithm performance were examined:

- Accuracy of dead-reckoning navigation
- Bias estimation process should return similar values for car stops that are close in time, but for which the elevation angle is different

**Figure 15.**
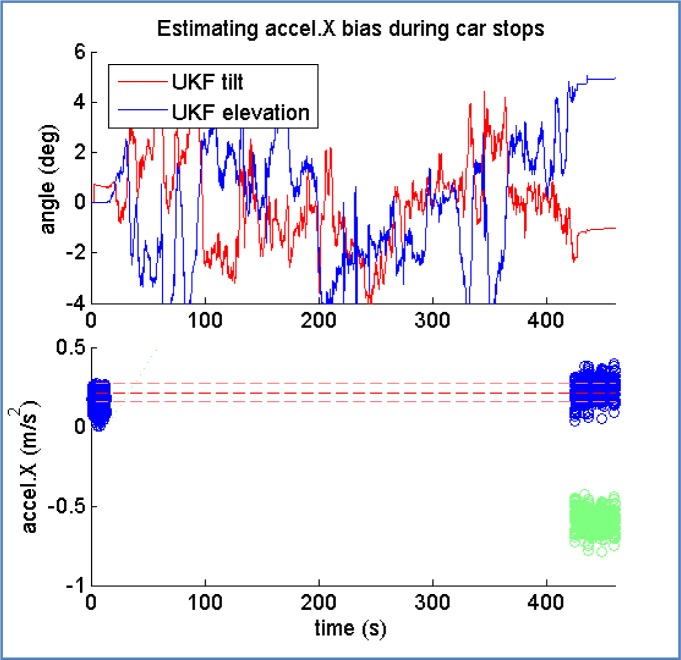
Accelerometer bias can be corrected during stops if elevation angle has been already determined.

The second point is illustrated in Figure 15, where the bias estimation process returns an average value of 0.21 m/s^2^ (blue circles in lower part) after correcting the effect of gravity according to the estimated car elevation, which is close to zero in the first stop and close to 5° in the second one. The green circles during the second stop represent the raw accelerometer reading, before correcting the gravity effect.

Regarding the estimation of car elevation angle from GPS positions the expected error, which depends on the random position error of the two consecutive GPS fixes, is difficult to describe analytically, and is clearly not distributed as a Gaussian. The selected solution involved a Monte Carlo simulation that describes the probability distribution function of the error. Its second order statistic (variance) was calculated, for getting an approximate Gaussian description of such error. The detailed procedure is described next:

- Input: two GPS measurements
- Calculate distance in the plane, difference in altitude, average moving speed, expected elevation angle
- Repeat N times (Monte Carlo):
  ○ Simulate real position of the vehicle for the input GPS fixes: add random sample distributed as a Gaussian described by GPS accuracy indicators
  ○ Calculate real elevation angle
  ○ Store the difference between expected and real elevation angle
- Calculate statistics for the distribution of the error of estimated elevation angle

**Figure 16.**
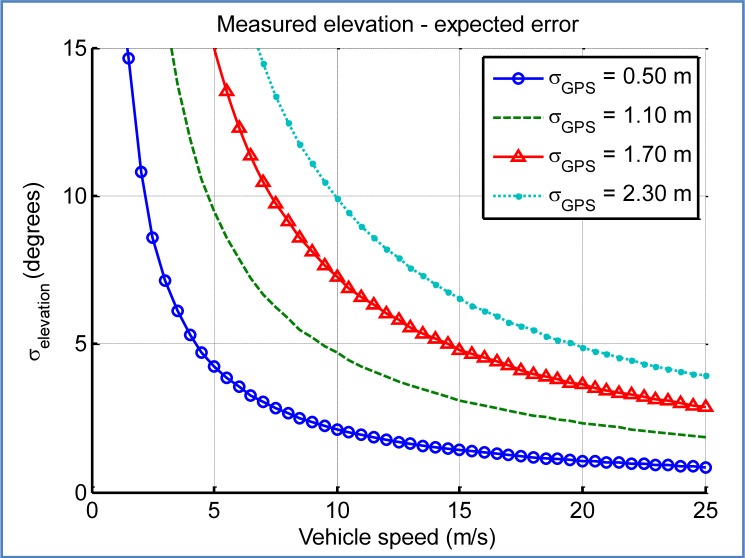
Expected standard deviation of GPS-obtained elevation angle, depending on vehicle speed and fix horizontal accuracy (simulation, 10 million iterations per point).

Figure 16 shows the expected standard deviation of the calculated elevation angle depending on the speed of the car and the accuracy of the GPS device. For a goal of degree-level accuracy, the conditions have to be near optimal, with a good GPS accuracy (standard deviation in the three axes around 1 m) and the car travelling at a high speed (over 10–15 m/s).

With respect to the whole navigation system, several complex scenarios have been selected to assess the overall performance. These experiments include typical cases as stops or turns in urban environments, enriched here with especially complex cases such as roundabouts with different exits, turns in the banked road at mountain pass, underground parking areas, long tunnels, driving under elevated pedestrian bridges, or short tunnels under motorways to change direction.

**Figure 17.**
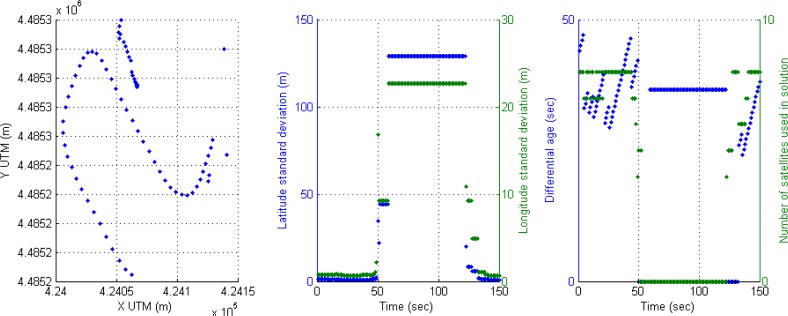
Non-underground parking area with zero satellite visibility and inactive DGPS mode using a constant value.

The first complex scenario includes a total GPS blackout in a non-underground parking. The calculated position by GPS appears as a constant value whereas the vehicle is passing through the parking area, which is not underground but has a roof that occludes the satellites (Figure 17). The standard deviations show a high value in the middle of the graph corresponding to this situation of non-available solution at the rover receiver. The right graph displays this effect of inactive DGPS mode maintaining a constant value of differential age and zero satellites used in the solution, when the vehicle is passing through the non-underground parking. It can be observed at the parking exit that the receiver changes to SINGLE mode when the satellites are visible. The left graph displays a gap of the trajectory caused by non-calculation of the coordinates by the receiver that maintains the last calculation. The GPS blackout has a total length of 56 s.

At this point, and after more than 10 min running, the system has accurately determined biases. Dead-reckoning conditions are not optimal, though, since at this point the last effective measure of the pitch was received more than two minutes ago, so the attitude has been maintained by the filter integrating IMU measures.

**Figure 18.**
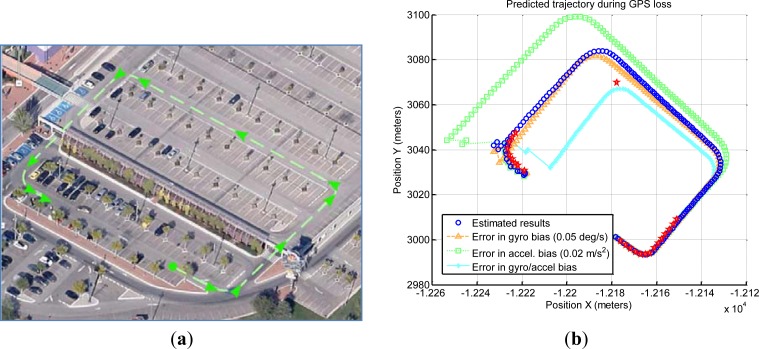
(**a**) Vehicle trajectory (**b**) Predicted trajectories.

Figure 18(b) shows that when GPS signal is recovered (red stars), the positioning error of the filter is around 15 m (blue circles). For establishing a comparison, three other predictions are shown, corresponding to simpler solutions where the sensors are not dynamically adjusted. They use IMU bias estimation with an error around 0.05 degrees per second for the gyroscope and/or 0.02 m/s^2^ for the accelerometer.

The estimation for gyroscope bias that the proposed system achieves is stable within 0.02 degrees per second. The error in position caused by the drifting attitude estimation is not very important compared with that of the accelerometer. It is reasonable to conclude that gyro bias estimation is accurate enough in our system. It is different for the accelerometer bias, where an error of 0.2 m/s^2^ has a much profound impact. It is worth remembering that residual accelerations of a similar magnitude can appear spontaneously if the vehicle elevation is estimated with a deviation of 1 degree.

In conclusion, the results on this scenario show that the biases estimated by the proposed system have been set correctly, and that small changes inside the expected IMU bias stability can be the source of large errors.

**Figure 19.**
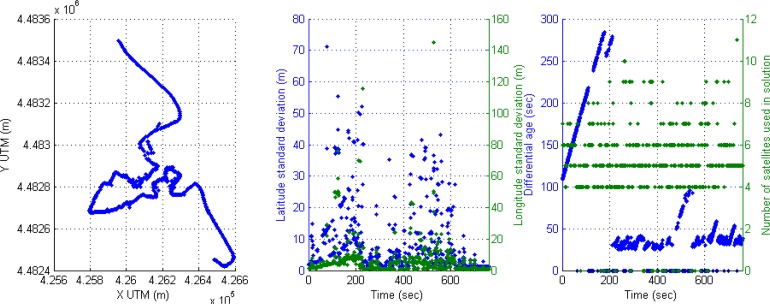
Complex urban canyons with a plethora of cases with non-optimal conditions for the rover receiver.

The second scenario is related to a complex urban environment where the vehicle is passing through urban canyons with low visibility of satellites. The Figure 19 shows cases with active DGPS mode, cases with active DGPS and high values of differential ages, cases with inactive DGPS mode and active SINGLE mode solution, and cases with zero satellites used in solution. The trajectory can be observed in the left graph where the vehicle arrives to complex urban canyons, and the rover receiver is changing frequently their mode depending on environment conditions through complex urban environment. The accuracy is recovered at the exit of the urban canyon and this effect is detected at the end of the middle graph. The right graph displays the diversity of cases presented in this experiment, thus it is difficult to obtain optimal conditions in complex urban canyons that can be solved by sensor fusion.

**Figure 20.**
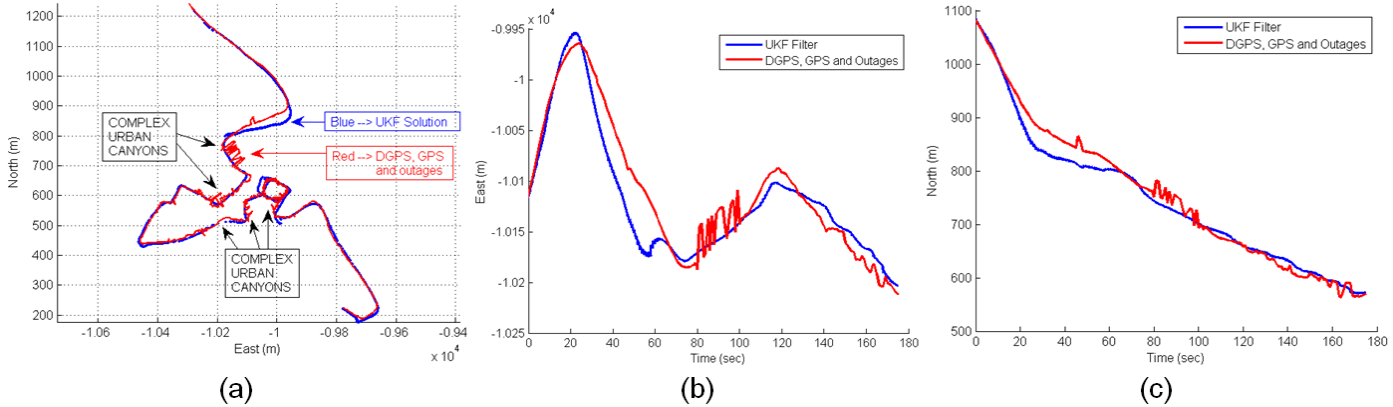
(**a**) Rover trajectories within complex urban canyons: DGPS, GPS and Outages (red), and UKF solution (blue) (**b**) Time-domain detail of East UKF solution. (**c**) Time-domain detail of North UKF solution.

The sensor fusion solution is presented in Figure 20(a), where red trajectory displays the difficult calculation of positioning by rover DGPS system. Inaccuracies are caused by rover navigation within complex urban area. The estimated solution using UKF filter is blue trajectory. The reliability of UKF solution can be observed in detail for 175 s of initial trajectory: Figure 20(b), East coordinate and Figure 20(c), North coordinate.

**Figure 21.**
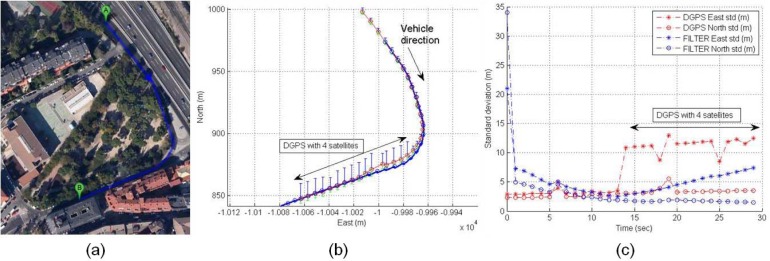
(**a**) Rover trajectory entering in an urban area. (**b**) Loss of accuracy in a soft urban environment (DGPS with four satellites) and UKF filter solution. (**c**) Time-domain standard deviation evolutions (DGPS and filter).

The effect of entering an urban area is displayed in Figure 21(a). Initially the DGPS trajectory is the same that the UKF filter trajectory, but DGPS inaccuracy appears when the rover is close to big trees and is entering a soft urban environment. Figure 21(b) displays an increase of DGPS East standard deviation caused to use four satellites in the solution with high differential ages, and Figure 21(c) is the time-domain representation of the standard deviations where the filter shows low positioning errors.

**Figure 22.**
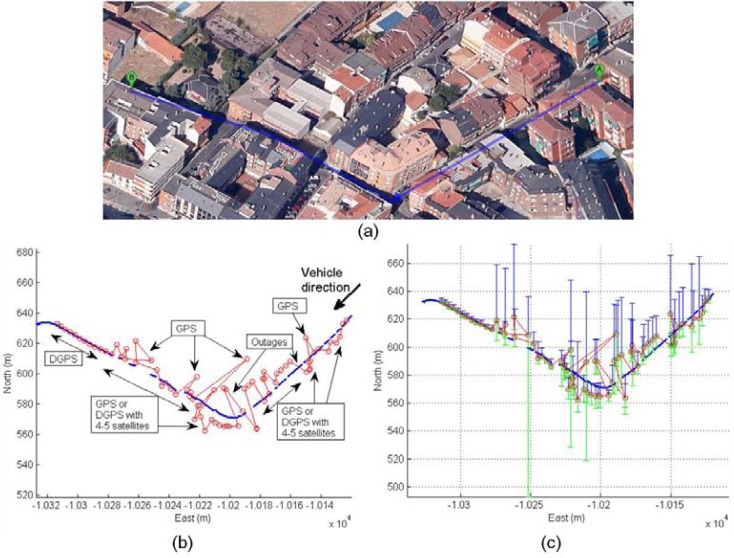
(**a**) Rover trajectory within complex urban canyon. (**b**) DGPS and GPS solutions (red) and UKF filter solution (blue). (**c**) Standard deviations of DGPS and GPS: East (upper blue bar) and North (lower green bar).

The movement of the vehicle in a complex canyon is displayed in Figure 22(a), where close buildings cause GPS and DGPS inaccuracies, and outages. The UKF solution is presented in Figure 22(b) (blue trajectory), and shows the filter reliability with a smooth trajectory that corresponds to the real trajectory following by the vehicle, as can be observed in Figure 22(a). The GPS and DGPS standard deviations are presented in Figure 22(c) to show the positioning errors that are solved by sensor fusion.

**Figure 23.**
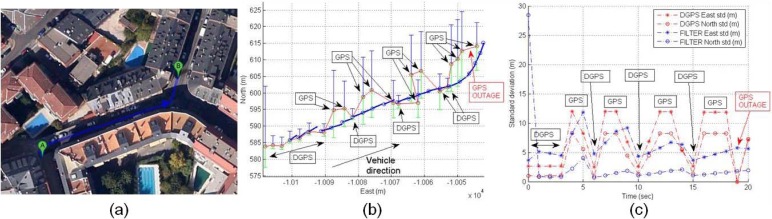
(**a**) Second rover trajectory within complex urban canyon. (**b**) DGPS and GPS solutions (red) and UKF filter solution (blue). (**c**) Time-domain standard deviation evolutions (DGPS and filter).

A third situation is shown in Figure 23(a). This urban trajectory presents several inaccuracies and the UKF filter solution displays again reliability to estimate the position. The difference with the former case is the use of the filter solution only for this trajectory, so the filter is started at the beginning of this trajectory. The estimated positions by the filter are shown in Figure 23(b). In this case, the filter solution has again better precision than the GNSS device, and time-domain standard deviations of Figure 23(c) shows the performance of the filter.

**Figure 24.**
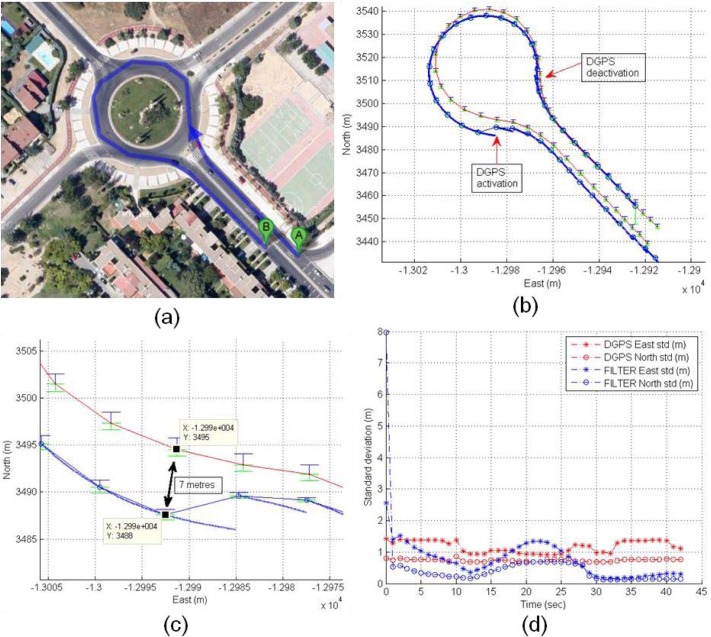
(**a**) Roundabout with DGPS groundtruth. (**b**) DGPS trajectory (red) with deactivation and activation and UKF filter solution (blue). (**c**) Detail of UKF filter solution without 15 seconds of DGPS solution. (**d**) Time-domain standard deviation evolutions.

The validation of the UKF filter solution is presented in Figure 24(a). The validation is based on the comparison of the DGPS precise trajectory as groundtruth with calculated solution provided by the UKF filter. Figure 24(b) displays the validation experiment, where DGPS deactivation is performed at the beginning of a roundabout and the reactivation is at the end of the roundabout, so the UKF filter has a deactivated GNSS positioning for 15 seconds. In terms of correlation, both coordinates presents good results, the R^2^ values for East and North coordinates are 0.9959 and 0.9904. The deviation of the real trajectory at the end of roundabout is 7 m as can be observed in Figure 24(c), where the East standard deviations (DGPS and filter) are indicated as upper blue bar, and North as lower green bar. The time-domain standard deviations of DGPS and filter are compared showing an increase of the UKF filter errors from second 12 to second 27 (Figure 24(d)), the effect corresponds to deactivation and reactivation of DGPS device.

## 6. Conclusions

In this article, vehicle positioning has been studied as a complex and essential task for Intelligent Transportation Systems in urban environments. A reliable solution based on a context-aided Unscented Kalman Filter has been proposed by fusing Differential Global Positioning System and Inertial Measurement Unit to estimate the vehicle positioning. A context-aided module aids the non-linear estimation process, with an explicit representation and inference about contextual situation. The usefulness of the presented system, and comparative advantages with respect to simpler approaches, have been extensively demonstrated through results under demanding circumstances such as GPS outages, degraded satellite signals, loss of differential mode or multi-path presence, while maintaining the positioning accuracy in complex urban scenarios.

Complex scenarios in urban environments ranging from non-underground parking to urban canyon trajectories, have been analyzed, and former GPS difficulties have been be overcome successfully. The time-domain comparison of GPS/DGPS and UKF filter solutions has corroborated the optimal UKF filter solution in different urban trajectories. The first validation of the advanced GNSS/IMU fusion system has been quantified through the comparisons of standard deviation evolutions of GPS/DGPS and UKF filter solutions. The second validation of the UKF filter solution have been demonstrated by means of the comparison between high-accuracy DGPS groundtruth and UKF filter trajectory.

This solution can be applied, using moderate-cost available sensors, in forthcoming vehicles that will require reliable positioning in urban environments, such as cooperative driving, automatic maneuvers for pedestrian safety, autonomous urban vehicles, and collision avoidance, among other ITS applications.

## Figures and Tables

**Table 1.** Filtering algorithm for determining vehicle attitude.

**Initialization**		Define state vector ***x*_0_** = [***ψ***, ***ϕ***]*^T^* Define initial covariance matrix $P_{0}=\left[\begin{array}{cc} \sigma_{\psi } & 0\\ 0 & \sigma_{\phi } \end{array}\right]$
**Prediction function** **Prediction *x_k+1_* = *f*(*x_k_*, *u_k_*, Δ*t*)**		For gyroscope reading *u_k_* = [*g_x_*, *g_y_*, *g_z_*]*^T^*: Transform vehicle attitude *x_k_* to matrix form *M*(*x*)*_k_* Calculate total rotation matrix *M_rot_* for angular rate *u_k_* and time increment Δ*t* Calculate new predicted attitude *M*(*x*)*_k_*_+1_ = *M*(*x*)*_k_* · *M_rot_*. Transform to Euler tern and extract tilt/elevation: *x_k_*_+1_ = [*ψ*′, ϕ′]*^T^*
**Update**	Tilt	In the arrival of a tilt measure: Set matrix *H* = [1 0] as measure function (it is linear) Set observation covariance as *Q* = *σ_tilt_*, where *σ_tilt_* is a value determined by accelerometer noise. Acceptable values are in the range [0.3; 1] degrees.
	Elevation	In the arrival of an elevation measure: Set matrix *H* = [0 1] as measure function Determine the expected measure error: ○ Take vehicle speed ○ Take accuracy of the GPS fixes used to calculate the elevation measure ○ Using those two values, retrieve the expected elevation measure error *σ_elev_* from the lookup table (details on following subsections) Set observation covariance as *Q = σ_elev_*

## References

[1] (2007). Concept of Operations for the Next Generation Air Transportation System.

[2] El Faouzi N.-E., Leung H., Kurian A. (2011). Data fusion in intelligent transportation systems: Progress and challenges—A survey. Inform. Fusion.

[3] DARPA Challenges.

[4] DARPA Urban Challenge.

[5] Urmson C., Anhalt J., Bagnell D., Baker C., Bittner R., Clark M.N., Dolan J., Duggins D., Galatali T., Geyer C. (2008). Autonomous driving in urban environments: Boss and the urban challenge. J. Field Robot.

[6] Nastro L. Position and Orientation Data Requirements for Precise Autonomous Vehicle Navigation.

[7] Shladover S.E. (2009). Cooperative (Rather than Autonomous) vehicle highway automation systems. IEEE Intel. Transp. Syst. Mag.

[8] Sengupta R., Rezaei S., Shladover S.E., Misener J.A., Dickey S., Krishnan H. (2007). Cooperative collision warning systems: Concept definition and experimental implementation. J. Intell. Transport. Syst.

[9] Morrison A., Renaudin V., Bancroft J.B., Lachapelle G. (2012). Design and testing of a multi-sensor pedestrian location and navigation platform. Sensors.

[10] Chae H., Christiand, Choi S., Yu W., Cho J. Autonomous Navigation of Mobile Robot based on DGPS/INS Sensor Fusion by EKF in Semi-outdoor Structured Environment.

[11] Morales Y., Takeuchi E., Tsubouchi T. Vehicle Localization in Outdoor Woodland Environments with Sensor Fault Detection.

[12] Britting K.R. (2010). Inertial Navigation Systems Analysis.

[13] Caron F., Duflos E., Pomorski D., Vanheeghe P. (2006). GPS/IMU data fusion using multisensor Kalman Filtering: Introduction of contextual aspects. Inform. Fusion.

[14] Corrales J.A., Candelas F.A., Torres F. (2010). Sensor data integration for indoor human tracking. Robot. Auton. Syst.

[15] Van der Merwe R., Wan E., Julier S. Sigma Point Kalman Filters for Nonlinear Estimation and Sensor Fusion: Applications to Integrated Navigation.

[16] Hajiyev C., Tutucu M.A. Development of GPS Aided INS via Federated Kalman Filter.

[17] Carlson N.A. Federated Filter for Distributed Navigation and Tracking Applications.

[18] Eldredge A.M. (2006). Improved State Estimation for Miniature Air Vehicles.

[19] Wagner J.F. (2005). GNSS/INS integration: still an attractive candidate for automatic landing systems?. GPS Sol.

[20] Cheng Y., Crassidis J.L. Particle Filtering for Attitude Estimation Using a Computational Efficient Quaternion Representation.

[21] Vernaza P., Lee D.D., Khatib O., Kumar V., Rus D. (2008). Robust GPS/INS-Aided Localization and Mapping via GPS Bias Estimation. Springer Tracts in Advanced Robotics, Experimental Robotics.

[22] Coopmans C., Chao H., Chen Y.Q. Design and Implementation of Sensing and Estimation Software in AGGIENAV, a Small UAV Navigation Platform.

[23] Crassidis J.L., Markley F.L. (2003). Unscented Filtering for Spacecraft Attitude Estimation. AIAA J. Guid. Control. Dyn.

[24] Zhou J., Knedlik S., Loffeld O. (2010). INS/GPS Tightly-coupled Integration using Adaptive Unscented Particle Filter. J. Navig.

[25] Li W, Leung H. Constrained Unscented Kalman Filter Based Fusion of GPS/INS/Digital Map for Vehicle Localization.

[26] Nordlund P.-J., Gustafsson F. (2009). Marginalized particle filter for accurate and reliable terrain-aided navigation. IEEE Trans. Aerosp. Electron. Syst.

[27] Blom H.A.P., Bar-Shalom Y. (1988). The interacting multiple model algorithm for systems with marcovian switching coefficients. IEEE Trans. Autom. Control.

[28] Ndjeng Ndjena A., Gruyer D., Glaser S., Lambert A. (2011). Low Cost IMU–Odometer–GPS Ego Localization for Unusual Maneuvers. Inform. Fusion.

[29] Rezaei S., Sengupta R. (2007). Kalman Filter-Based Integration of DGPS and Vehicle Sensors for Localization. IEEE Trans. Control Syst. Technol.

[30] Soloviev A. Tight Coupling of GPS, Laser Scanner, and Inertial Measurements for Navigation in Urban Environments.

[31] García J., Berlanga A., Molina J.M. (2009). Effective evolutionary algorithms for multiple specifications attainment. Application to design of ATC tracking filters. IEEE Trans. Evol. Computat.

[32] Noureldin A., El-Shafie A., Bayoumi M. (2011). GPS/INS Integration utilizing dynamic neural networks for vehicular navigation. Inform. Fusion.

[33] Belkin B.L., Stengel R.F. (1990). Quantitative knowledge acquisition for expert systems. Eng. Appl. Artif. Intell.

[34] Wang J-H., Gao Y. (2007). The aiding of MEMS INS/GPS integration using artificial intelligence for land vehicle navigation. IAENG Int. J. Comput. Sci..

[35] Ulmke M., Koch W. (2006). Road-Map Assisted Ground Moving Target Tracking. IEEE Trans. Aerosp. Electron. Syst.

[36] Pannetier B., Benameur K., Nimier V., Rombaut M. VS-IMM Using Road Map Information for a Ground Target Tracking.

[37] Martí E.D., García J., Crassidis J.L. Improving Multiple-Model Context-Aided Tracking through an Autocorrelation Approach.

[38] Semerdjiev E., Mihaylova L. (2000). Variable- and fixed-structure augmented interacting multiple model algorithms for manoeuvring ship tracking based on new ship models. Int. J. Appl. Math.Comput. Sci.

[39] Powell G.M., Matheus C.J., Kokar M.M., Lorenz D. Understanding the Role of Context in the Interpretation of Complex Battlespace Intelligence.

[40] Bosse E., Valin P., Boury-Brisset A.-C., Grenier D. (2006). Exploitation of *a priori* knowledge for information fusion. Inform. Fusion.

[41] Antony R.T., Karakowski J.A. Towards Greater Consciousness in Data Fusion Systems.

[42] Garcia J., Gomez-Romero J., Patricio M.A., Molina J.M., Rogova G.L. On the Representation and Exploitation of Context Knowledge in a Harbor Surveillance Scenario.

[43] Fakharian A., Gustafsson T., Mehrfam M. Adaptive Kalman Filtering Based Navigation: An IMU/GPS Integration Approach.

[44] NovAtel Inc. OEMV Family of Receivers—Firmware Reference Manual. Publication Number: OM-20000094; Available online: www.novatel.com (accessed on 22 August 2012).

[45] Grewal M.S., Weill L.R., Andrews A.P. (2007). Global Positioning Systems, Inertial Navigation, and Integration.

[46] Lee D., Lee S., Park S., Ko S. (2011). Test and Error Parameter Estimation for MEMS—Based Low Cost IMU Calibration. Int. J. Precis. Eng. Manuf.

[47] Frigo M., Johnson S.G. FFTW: An Adaptive Software Architecture for the FFT.

[48] Hafner P., Wieser M., Kühtreiber N. (2011). Quality Assessment of Different GNSS/IMS-Integrations. Österr. Z. Vermess. u. Geo-Inform.

[49] Zhang P., Gu J., Milios E.E., Huynh P. Navigation with IMU/GPS/Digital Compass with Unscented Kalman Filter.

[50] Martí E.D., García J., Molina J.M. Context-Awareness at the Service of Sensor Fusion Systems: Inverting the Usual Scheme.

[51] Welch G., Bishop G. (1995). An Introduction to the Kalman Filter.

[52] Julier S.J., Uhlmann J.K. A New Extension of the Kalman Filter to Nonlinear Systems.

[53] Van der Merwe R., Doucet A., de Freitas N., Wan E. (2000). The Unscented Particle Filter. Advances in Neural Information Processing Systems (NIPS13).

[54] Dwiggins B.H. (1968). Automotive Steering Systems.

[55] Mandapat R.E. (2001). Development and Evaluation of Positioning Systems for Autonomous Vehicle Navigation.

